# Why Coagulants Diffuse through Dentine

**Published:** 1898-09

**Authors:** E. Lawley York

**Affiliations:** England


### Abstracts and Translations.

### WHY COAGULANTS DIFFUSE THROUGH DENTINE.

BY E. LAWLEY YORK, D.D.S., F.R.M.S., ENGLAND.[1]

About a year ago I had the honor to present to this society a paper on "The Diffusibility of Coagulants in Dentine." The deductions I drew and the experiments I exhibited at the time

[1] Bacteriologist in Chicago College of Dental Surgery, Chicago, Ill.

showed you conclusively that carbolic acid would diffuse through dentine. Hearing that I was continuing this line of investigation, the chairman of your Executive Committee did me the honor to request me to read another paper along the same lines, and from the generous and kindly manner in which you received my former effort, I consented. The following is the result of my experiments extending over the past year.

If you remember, I stated at that time that I found that there was greater rapidity in the diffusion of carbolic acid through the dentine of a tooth that had contained a putrescent pulp (four to eight hours). This occurred not once only, but in every case, and it set me thinking. Why was there greater rapidity of diffusion in such a tooth and less in one that had contained up to the time of treatment a normal pulp? In the latter we had to deal with a tooth that had so far not undergone any pathological changes, either in the contents of the pulp-chamber or the dentinal tubuli, all the albumen that is normally in a tooth being there intact, whereas in the tooth the pulp of which had died, and as a consequence undergone the process of putrefaction and the consequent formation of an innumerable number of end-products, we had a totally different condition to deal with. In the first condition we might have an infinitesimal quantity of albumen to deal with, as I will show you later, whereas in the latter we have none, as I will now endeavor to demonstrate.

Many mouth-bacteria, as well as the majority of the pyogenic and putrefactive bacteria, have the faculty of dissolving coagulated albumen or albuminous substances, of peptonizing or converting them into soluble substances, just as albumen is converted into soluble peptones by the pepsin of the gastric juice. Micro-organisms nourish themselves only by substances in a state of solution, and if we present them solid substances they must first liquefy these substances before they can make any use of them for their own nourishment.

After the death of a pulp it is invaded by various bacteria, strictly saprogenic as well as pathogenic, the result of which is that the pulp becomes a foul, semifluid mass. This putrefactive decomposition of albuminous matter is effected by a great variety of micro-organisms and gives rise to a great variety of products, some of which are volatile and are characterized by their offensive odors. This putrescence was the result of, first, the splitting up of the albumens into peptones, which according to Flugge may be effected by a number of micro-organisms, then the splitting up of the peptones into a large number of gases, acids, bases, and salts. Among the products of putrefactive fermentation known to chemists are the following: Carbon dioxide, hydrogen, nitrogen, hydrosulphuric acid, phosphuretted hydrogen, methane, formic acid, acetic acid, butyric acid, valerianic acid, palmitic acid, crotonic acid, etc. A few words on ptomaines may not be out of place. It is a name suggested by the Italian toxicologist, Selmi, and derived from the Greek word πτωμα, meaning a cadaver.

A ptomaine may be defined as an organic chemical compound, basic in character, and formed by the action of bacteria on nitrogenous matter. They have also been called animal alkaloids, but this is a misnomer, because, in the first place, some of them have been found in the putrefaction of vegetable matter, and in the second place, the term animal alkaloid is more properly restricted to the leucomaines, those basic substances which result from tissue metabolism. While some of the ptomaines are highly poisonous, this is not an essential property, and others are entirely inert. Hence the severe and complicated conditions following in some cases a blind abscess, or the opening of a putrescent pulp-canal, where we have exercised the greatest care. Since all putrefaction is due to the action of bacteria, it follows that all ptomaines result from the growth of these organisms. The kind of ptomaine formed will depend upon the individual bacterium engaged in its production, the nature of the material being acted upon, and the conditions under which the putrefaction goes on, such as the temperature, the amount of oxygen present, and the duration of the process. Ptomaines are the transition products in the process of putrefaction. They are temporary forms through which matter passes while it is being transformed by the activity of bacterial life from the organic to the inorganic state. Complex organic substances, such as muscle and brain, are broken up into less complex molecules, and so the process of chemic division goes on until the simple and well-known final products, carbonic acid, ammonia, and water, result.

It is an established fact, and will be borne out by the experiments which I will give you later, that the end-products of albumen decomposition, or putrefaction, are no longer coagulable. I previously stated in this paper that the first step in the process of putrefaction is the transformation of the albumens into peptones. Now these peptones are not coagulable; for example, if you take pepsin and add it to serum albumen and allow it to digest at body temperature, you will find it is converted into peptones, etc., which are not coagulable. This is precisely the same condition that we find produced by the action of peptonizing bacteria upon proteid matter. (Here exhibit tubes of decomposed serum albumen, and tubes of serum albumen to which has been added pepsin.) On the addition of carbolic acid they do not coagulate.

Now, how does carbolic acid act upon these substances? Does it coagulate the orificial ends of the dentinal tubuli, and seal in all this poisonous matter? Most emphatically no. The carbolic acid will penetrate as well as anything else you may use.

Let us now take another view of this much mooted question and see how the carbolic acid will act in the dentine of a tooth in which you have removed a normal pulp, one in which the albumen has not undergone decomposition. I stated earlier in my paper that carbolic acid diffused through the dentine of a tooth from which I had removed a normal pulp a trifle slower than one which contained a putrescent pulp. The reason for that was this: that carbolic acid did coagulate the trace of albumen that was there, but the former (carbolic acid) being in excess, the coagulum was redissolved. I will now show you capillary tubes filled with serum albumen (human), native albumen, and artificial serum albumen, and you will notice the coagulation proceeds slowly, and, following behind a trifle slower, you will see that the coagulum is being redissolved. This is precisely the thing that occurs in the dentinal tubuli, only we have such a minute quantity of albumen in the tooth-structure that it is hardly a factor.

To demonstrate the latter statement to your satisfaction I will give in detail some experiments made to determine the quantity of albumen in a tooth.

*Determination of Albumen in Teeth.*—The teeth are first thoroughly scraped, removing as much of the adhering particles as possible. They are then carefully brushed with alcohol, which coagulates the albumen on exterior of the teeth. After the teeth are dry they are finely pulverized.

To this pulverized substance (about ten grammes) is added a decinormal sodium chloride solution (about twenty-five cubic centimetres) alkalinized with sodium carbonate. This is thoroughly agitated and allowed to stand for thirty-six hours. During this time the mixture is frequently shaken. It is then filtered until a clear filtrate is obtained, and washed. The filtrate is acidified with dilute acetic acid and brought to the boiling-point. This coagulates the albumen. It is allowed to stand for some hours until the coagulum settles and the particles become agglutinated. It is then filtered upon a counterpoise filter. The precipitate is then washed until no reaction occurs upon the addition of a solution of silver nitrate.

The contents of the filter are then dried at a temperature of 110° C. for about thirty minutes, then placed in a desiccator and afterwards weighed. The albumen is repeatedly dried until a constant weight is obtained.

The tooth-substance, after being treated as above, was again subjected to the same process, but yielded only a faint trace of albumen, showing that practically all the albumen had been removed.

7.5 grammes (115.74 grains) yield .0028 gramme ($\frac{7}{160}$ grain), $\frac{37}{1000}$ of one per cent.

9.5 grammes (146.60 grains) yield .0067 gramme ($\frac{67}{640}$ grain), $\frac{7}{100}$ of one per cent.

11.5 grammes (177.46 grains) yield .0060 gramme ($\frac{3}{32}$ grain), $\frac{5}{100}$ of one per cent.

(1) These analyses of the teeth will clearly show you that the amount of albumen in a tooth is of too minute a quantity to be a factor. This applies to a tooth the pulp of which was in a normal condition when analyzed. (2) A tooth the pulp of which has undergone the process of putrefaction of albumen decomposition. The end-products are no longer coagulable. (3) Had we as large an amount of albumen in a normal tooth as we have always been led to believe, the quantity of carbolic acid which would be accommodated in the pulp-chamber and canals would be quite sufficient to redissolve any coagulum that would be formed.

*Recapitulation.*—I have shown you capillary tubes containing egg or native albumen, serum albumen (human), and artificial albumen, all of which coagulate in the presence of carbolic acid, and you will also observe redissolve in an excess of carbolic acid. None of these have undergone decomposition. I have also shown you capillary tubes filled with decomposed serum albumen (human), and gelatin and serum albumen (human), acted upon by various pathogenic and mouth bacteria, none of which show any sign of coagulating in the presence of carbolic acid.

After getting these uniform results by repeated experiments hundred of times, and drawing my own deductions from them, I was naturally anxious to communicate with others who might have been over similar ground in search of other subjects. So I accordingly opened up a correspondence with Professor Vaughn, of Ann Arbor, on the decomposition of albuminous substances, and will give you his reply: "There can be no doubt that the end-products of albumen decomposition are no longer coagulable."

Professor Klebs, of world-wide reputation, also states that they are no longer coagulable, as also does Professor Hektoen, of the bacteriological and pathological laboratories of the Rush Medical College.

Before closing, I have to thank those gentlemen for their courtesy in answering my questions, and also to express my indebtedness to Mr. O. T. Roberg, of the chemical laboratory of the Rush Medical College, for his valuable assistance in making the quantitative analyses of tooth-substances.

The books I have consulted and freely quoted from are "Ptomaines, Toxins and Antitoxins," by Vaughn and Novy; "Micro-organisms of the Human Mouth," Miller; McFarland's "Bacteriology;" and Sternberg's "Bacteriology."—*The Dental Review.*

